# Fun moments or consequential experiences? A model for conceptualising and researching equitable youth outcomes from informal STEM learning

**DOI:** 10.1007/s11422-021-10065-5

**Published:** 2022-02-25

**Authors:** Louise Archer, Angela M. Calabrese Barton, Emily Dawson, Spela Godec, Ada Mau, Uma Patel

**Affiliations:** University College London, Institute of Education, London, UK

**Keywords:** Equity, ISL, Youth, Outcomes

## Abstract

While there are many different frameworks seeking to identify what benefits young people might derive from participation in informal STEM (Science, Technology, Engineering and Mathematics) learning (ISL), this paper argues that the sector would benefit from an approach that foregrounds equity and social justice outcomes. We propose a new model for reflecting on equitable youth outcomes from ISL that identifies five key areas: (1) Grounded fun; (2) STEM capital; (3) STEM trajectories; (4) STEM identity work; and (5) Agency+ . The model is applied to empirical data (interviews, observations and youth portfolios) collected over one year in four UK-based ISL settings with 33 young people (aged 11–14), largely from communities that are traditionally under-represented in STEM. Analysis considers the extent to which participating youth experienced equitable outcomes, or not, in relation to the five areas. The paper concludes with a discussion of implications for ISL and how the model might support ongoing efforts to reimagine ISL as vehicle for social justice.

## Why does equity in ISL matter?

Learning does not just take place in formal settings, such as schools and universities. There is considerable academic, practitioner and policy interest in understanding and identifying the outcomes and impact of learning that takes place in a wide range of informal settings, such as cultural organisations, community groups and everyday life. In this paper, we are interested in issues of equity and social justice pertaining to ISL settings—that is, informal Science, Technology, Engineering and Mathematics learning settings, such as science centres, zoos and community STEM clubs. We use the terminology of equity, as opposed to equality, to signal a move away from notions of ‘treating everyone the same’ (which is sometimes synonymous with the term equality) towards a principle of providing differential treatment according to need. The concept of social justice takes these ideas a step further, calling for the disruption and transformation of systems and power relations that create and sustain relations of inequity and injustice.

There is particular policy interest in ISL, reflecting widespread concerns about how to increase and diversify STEM participation for societal economic growth and prosperity and to ensure active citizenship in contemporary technologically advanced societies. Indeed, as noted by numerous research analyses and policy reports such as the National Science Foundation’s Science and Engineering Indicators (2018) in the US and Emma Smith’s (2011) analyses of UK national data, girls, women, minority ethnic and working-class young people remain acutely under-represented in post-compulsory science, but particularly in the physical sciences and engineering. However, there is also recognition of how more could usefully be done to support young people to learn and engage STEM in ways that are valuable to them and their communities—not only towards future careers, but also in everyday life. We are particularly concerned with the kinds of learning that matter to young people and that make possible more equitable forms and patterns of engagement in STEM across settings and over time.

It has been widely argued, as exemplified by the work of John Falk and Lynn Dierking (2010) that ISL settings can provide an entry point for young people who have not previously been engaged by STEM learning at school. As discussed further below, there is also a body of research conducted in innovative, equity-focused out-of-school contexts which shows that ISL participation, particularly when youth are involved in long-term, community-based programmes, can support a range of outcomes for participating youth. However, as William Penuel (2017) notes, these have not been widely adopted across the sector and evidence demonstrates that in most ISL settings, participants tend to come from socially privileged, white, affluent backgrounds. Studies of science museums, such as the ethnographic work conducted by Emily Dawson (2014), reveal a range of culturally exclusionary practices and processes enacted in these spaces that act as barriers to both entry and participation. As Noah Feinstein and David Meshoulam (2014) argue, many ISL spaces tend to represent and reproduce dominant white, male, middle-class values, histories and identities. As a result, as found by Louise Archer, Emily Dawson, Amy Seakins and Billy Wong (2016a), minoritized youth and their families can experience these spaces as disorientating and alienating. The equitable potential of many informal STEM learning settings therefore appears to be currently under-developed and constrained, raising questions as to what benefits different young people derive from ISL experiences. Indeed, it has been argued that some ISL designed spaces, such as science museums, ‘almost certainly make inequities worse’ (Feinstein, 2017, p. 533).

Leslie Herrenkohl and Bronwyn Bevan (2017, p. 519) argue that ‘contexts outside of school are positioned to play a critical role in successfully addressing equity in STEM learning, but […] much deeper theorization, reflection, and conceptualization is needed to realize this potential’. In this paper, we focus on conceptualising and understanding the outcomes that urban young people aged 11–14, in particular those from communities traditionally under-represented and excluded from ISL, might gain from participation and the extent to which these outcomes might be equitable. In particular, we seek to add to existing knowledge by contributing (1) proposing an equitable outcomes model for understanding (equitable) youth outcomes from ISL (2) empirical explication of the outcomes model, using data from four ISL settings, and (3) implications from our analyses that might further support equitable ISL practice with young people from under-served communities. To these ends, we ask:

- How might we usefully conceptualise and capture equitable youth outcomes from ISL?

- To what extent did youth on the four focal ISL programmes experience equitable outcomes, or not, as a result of their participation?

## What difference does it make? Foregrounding equitable outcomes from ISL

An outcome can be defined as a result, effect or a consequence arising from an action or situation. For instance, the Miriam Webster online dictionary defines an outcome as ‘something that follows as a result or consequence’. Educational settings have long been examined, evaluated and held to account for the extent to which they can produce particular outcomes, but particularly those associated with learning. Within the formal educational sector, schools, colleges and universities are routinely required to report, and are scrutinised for, their capacity to produce forms of academic attainment and learning outcomes. Standardised measures of science attainment constitute the most common, high-stakes outcome for which the formal science education sector is held to account. However, while ISL settings are also often required (e.g. as a condition of funding) to capture and evaluate the outcomes that participants might derive from participation, these measures tend to be more diverse than those in formal education, capturing a wider range of outcomes (beyond learning, as assessed through measures of attainment) and are far less standardised.

Our aim in this paper is *not* to create a new comprehensive ISL outcomes model to replace existing measures. Rather, we attempt to develop a useable framework that has a distinct and intentional equity focus in order to help ISL practitioners to consider and reflect on issues pertaining to social justice. We envisage the framework as a tool that could be used alongside whatever other outcome measures a setting may choose (or be required) to use. We believe that a distinct focus on equitable outcomes is both useful and necessary for a number of reasons. First, equity issues appear to be marginal or absent within many of the existing common outcome instruments that are used by the sector. Second, many of our practice partners wanted a tool to help them better capture the equitable outcomes that they felt some of the young people on their programmes experienced as a result of participation. Third, our previous research highlighted that many ISL practitioners feel that they lack expertise and capacity for addressing issues of equity and social justice (e.g. Archer et al., 2016b). Finally, we are aware that at policy levels, the adage ‘what gets measured gets valued’ has a continued resonance.

So what do we mean by equitable outcomes? In this paper we take a critical and sociological perspective that identifies and understands an equitable youth outcome from ISL as one that results in:

- the disruption and restructuring of dominant power relations, forms of representation and distributions of resource and opportunity;
- meaningful support, benefits and/or enhancements to identities, capital, agency, trajectories, lives and the rightful presence in STEM of youth from under-served communities.

In other words, equitable youth outcomes are those which specifically challenge dominant, inequitable power relations, practices and epistemologies, and redistribute (and recognise non-dominant) resources/capital, identities, opportunities, trajectories, agency and forms of representation to the benefit of under-served youth and those from communities historically under-represented in STEM. That is, equitable youth ISL outcomes disrupt normative fields (of science, ISL) and result in benefits for under-served youth and communities, including the rightful presence of under-served youth in STEM/ISL (Tan & Calabrese Barton, 2020). To paraphrase Calabrese Barton and Tan (2010, p. 5), rightful presence refers to a young person’s legitimate and legitimized membership in a learning community because of who they are (not who they are expected to be), where the practices of the community support a re-structuring of power dynamics towards more socially just ends, by making both injustice and social change visible.

Hence, ISL participation may be associated with various different potential outcomes, but not all of these will necessarily be equitable. For instance, some youth outcomes—such as those which augment the identities, knowledge and resources of youth from dominant communities who are traditionally over-represented in STEM and/or which bolster White, masculine, middle-class associations of STEM—may contribute to the reproduction of social inequalities and would not be counted as *equitable* outcomes, even if these experiences result in tangible benefits for those individuals involved.

## Developing a framework for conceptualising equitable youth outcomes from ISL

To develop a framework for capturing equitable youth outcomes from ISL, we drew on critical ISL and sociological conceptual lenses that foreground issues of complexity, power and injustice. We wanted to use approaches that engage with complexity because, as Bell et al. (2009) discuss, ISL outcomes are necessarily complex and can: encompass a broad range of behaviours, be unanticipated (not just expected or hoped for outcomes), become evident at different points in time and occur at different scales. Given our focus on trying to capture equitable youth outcomes, we also wanted to use conceptual lenses that fundamentally recognise and engage with issues of power, injustice and intersectionality and attend to the interplay of agency and structure. As is detailed further below in relation to each area of our model, the critical ISL literature is particularly apt in all these respects and our model is substantially informed by research and thinking from this body of work. Much of this research usefully and productively foregrounds agency, but in order to bring issues of structure also to the fore, we additionally drew on the sociology of Pierre Bourdieu (e.g. 1977). Bourdieu’s conceptual work is closely aligned with critical approaches in that it foregrounds issues of power, (in)justice and recognises the interplay of agency and structure in the production of social life. It also provides a complementary emphasis upon structural inequalities and the social reproduction of inequalities through interactions between young people and educational settings. Hence, we drew on Bourdieu’s notion of agency as both structured (by relations and experiences of power and injustice) and as structuring (that is, acting in turn on the field). Similar conceptualisations of the interplay between structure and agency are also found within various critical literatures (such as la paperson, 2017), which, drawing on foundational Black feminist thought (such as writings by Angela Davis, Audre Lorde and many more), draw attention to the important potential for structured agency (but particularly among those who are positioned through relations of exploitation, marginalisation and oppression) to move beyond existing conditions, to reimagine the world in more socially just ways. For instance, the critical ISL literature draws attention to the social justice potential of minortized youth’s reimaginings of society and STEM through their STEM-rich making (e.g. Calabrese Barton & Tan, 2018). In this way, the two complementary lenses strengthened our ability to attend to both structure and agency in relation to equitable youth outcomes from ISL.

Based on our conceptualisation of equitable outcomes and through our reading of the critical ISL literature, we initially identified five main equitable outcome areas: (1) STEM learning, skills, knowledge and funds of knowledge; (2) STEM attitudes and interests; (3) STEM path-making and progression (4) STEM identity and identity work and (5) Critical STEM agency. However, bringing in our sociological lens, we felt that the first two of these areas (STEM learning and STEM attitudes) could be conceptually combined through the notion of *STEM capital*. The sociological lens also informed the introduction of an additional outcome area—*grounded fun*—recognising that enjoyment/fun are areas that are commonly captured in many ISL outcome frameworks and which may have resonance for practitioners and which, according to our lens, also link to issues of identity and equity.

While our equity outcomes framework is conceptually derived, we also considered the extent to which the key five areas had some resonance with existing ‘traditional’ ISL outcomes frameworks. For instance, two of the most widely known and commonly used tools for capturing outcomes from ISL are, in the US, the NSF Framework for Evaluation Impacts of Informal Science Projects (2008), which specifies six main outcome areas (awareness, knowledge and understanding; engagement or interest; attitude; behaviour; skills; other) and in the UK, many arts, science and cultural organisations use the Generic Learning Outcomes (GLO) framework, which uses five key outcome areas (skills; knowledge and understanding; enjoyment, inspiration and creativity; attitudes and values; behaviour and progression). We also considered a range of other frameworks and publications, including reports by: the Afterschool Alliance (2011); Anita Krishnamurthi and Leonie Rennie’s (2013) wide-ranging review of informal science learning; John Falk, Lynn Dierking, Jonathan Osborne, Matthew Wenger, Emily Dawson and Billy Wong’s (2012) review of the contribution of the informal sector to UK science education; Phillip Bell, Bruce Lewenstein, Andrew Shouse and Michael Feder’s (2009) review of science learning in informal environments; and Richard Lloyd, Ross Nielson, Suzanne King and Mark Dyball’s (2012) review of ISL for the Wellcome Trust. We reviewed online outcomes models espoused by the Arts Council England, the National Research Council and the UK Museums, Libraries and Archives Council. From these, we noted that the following five categories seemed to be the most commonly measured and prioritised outcome areas: (1) fun/enjoyment; (2) STEM learning, knowledge and skills; (3) STEM attitudes, interest and inspiration (4) STEM aspirations; (5) twentyfirst century skills. As shown in Table 1, the first four of these have resonant areas with our own equitable outcomes model. There is no direct equivalent in our model to twentyfirst century skills, (those relating to critical thinking, creativity, collaboration, communication, Information/media/technology literacy, flexibility, leadership, initiative, productivity and social skills), although we suggest that some aspects of these may fall within STEM capital and Agency + .

**Table 1:** Situating the Equitable Youth Outcomes from ISL model

Common existing ‘traditional’ outcome measure aras	Critical ISL research	Bourdieusian (sociological) lens	Equitable youth outcomes from ISL Model
Fun/enjoyment	*'*Science that matters'	What and whose habitus is valued by the field	‘Grounded’ fun
STEM learning, knowledge & skills	STEM learning and skills, broadened/inclusive STEM knowledge and leveraging of funds of knowledge	STEM capital (knowledge, learning, literacies, use-value and exchange value capital)	STEM capital —STEM learning, literacy, skills, broadened/inclusive STEM knowledges and leveraging of funds of knowledge (use-value capital)
STEM interest, attitudes, inspiration	STEM interest and (broader/inclusive) attitudes	STEM habitus/capital	STEM capital—interest and (broader, inclusive) dispositions
		STEM capital—social capital	STEM capital—social contacts and networks
	STEM identity and identity work	STEM habitus	STEM identity/STEM identity work
STEM aspirations	STEM path-making, path-hacking and progression	Role of structural inequalities and field on habitus and trajectory	STEM trajectories
‘Twenty-first century skills’	Critical STEM agency, representation, rightful presence	Structured agency	Agency+ (Critical STEM agency, representation)

As detailed in Table 1, there was a considerable degree of overlap between many of the outcome areas, which gave us some reassurance that our proposed framework focusing on equitable youth outcomes was not entirely removed from the everyday interests and concerns of many ISL practitioners—although it provides a specific lens through which to interpret and reflect on common outcome areas. The rationale and detail for each of the five areas within the equitable outcomes model are now discussed in turn.

## Grounded fun

Outcomes relating to fun and enjoyment are commonly captured by ISL settings and providers. Even the shortest evaluation tools tend to ask how much a young person enjoyed an ISL experience and/or found it fun. Indeed, the review by Falk and colleagues (2012) found that ISL sectors and providers were almost unanimous in viewing an enjoyable ISL experience as an important outcome from ISL participation. However, from our conceptual position, a focus on fun/enjoyment per se can potentially hide the (in)equitable potential of an ISL experience. For instance, Daniel Birmingham and colleagues (writing in a 2014 article with Angela Calabrese Barton and a 2017 paper with Angela Calabrese Barton, Autumn McDaniel, Jalah Jones, Camryn Turner and Angel Rogers) discuss the value of fun STEM learning experiences being related to *science that matters* for under-served youth and communities.

Developing these ideas further, we suggest that equitable youth outcomes relating to fun and enjoyment need to be *grounded* if they to be of consequence—on its own, a fun experience does not necessarily equate with an equitable experience or outcome. Rather, the equitable potential of fun is realised when it is experienced in relation to something that young people themselves feel or recognise as being of interest, value or consequence to them. As Suzanne Hidi and Ann Renninger (2006) explain, an *interesting* experience implies a cognitive connection between an individual, their identity and the content or experience in question. We extend these ideas to suggest that grounded fun must involve some form of connection with the personal, authentic sense(s) of self that a young person values. The importance for an ISL experience to support and recognize a young person’s valued sense of self and identity work is underlined in the critical ISL literature (and is discussed further in the section of STEM identity work). From a sociological perspective, this point is explained as the reworking of what and whose habitus and capital is valued by the field—again underlining the importance of grounding fun in the habitus/identities, values and interests of young people if it is to help challenge rather than reproduce unequal power relations. Hence fun experiences of science may be either unconnected to, or even a distraction from, meaningful and consequential science learning that matters if it is not sufficiently grounded. In this respect, our model seeks to focus attention on capturing only young people’s experiences of meaningful fun and enjoyment, that is, those grounded in their identities (habitus) and what matters to them.

In sum, in our model, *grounded fun* refers to (1) youth having pleasurable, fun ISL experiences that are grounded in their identities and what matters to them (2) youth having enjoyable experiences of ISL that challenge dominant normative STEM relationships and representations. While we recognise that, strictly speaking, grounded fun may not be an outcome in quite the same way as the other outcomes areas in the model, we felt that due to its centrality in so much common ISL evaluation practice, there is a value to including it in the model.

## STEM capital (STEM learning, knowledge, skills and funds of knowledge)

As can commonly be found in most ISL evaluation instruments, ISL researchers and practitioners share an interest in outcomes relating to the development of STEM learning, knowledge, skills and dispositions among young people as a result of ISL participation. While STEM learning outcomes are frequently measured, concerns have been expressed that they often remain underspecified and are 'rarely articulated with clarity' (Krisnamurthi & Rennie 2013, p. 4, with similar criticisms voiced by Bell et al., 2009).

From our conceptual position, we are primarily interested in the extent to which STEM knowledge, learning and skills outcomes can be supported among under-served youth and 'can have a significant impact on science learning outcomes for individuals from non-dominant groups who are historically underrepresented in science' (Bell et al. 2009, p. 301). That is, from an equity standpoint, we are more concerned with *whose* STEM knowledge, skills and learning is being supported and *for whom* by an ISL experience rather than ‘how much’ someone has learned. Drawing on the work of Douglas Medin and Megan Bang (2014), our model recognises the role of power in structuring knowledges, asking *whose* knowledge are being recognised and legitimated (or devalued, rendered invisible or marginalised) in a particular context and the extent to which this STEM knowledge is consequential for youth and community agency and wellbeing. In this respect, our model does not seek to ‘measure’ what science learning or knowledge is gained, but rather seeks to support practitioners to consider the ways in which STEM knowledge is constructed and reconstructed within ISL programmes and to what ends. In other words, the model values a restructuring of dominant STEM knowledges and epistemologies towards more inclusive knowledges in addition to recognizing the extent to which under-served young people’s funds of knowledge are recognized, valued and leveraged within ISL. As Bronwyn Bevan, Angela Calabrese Barton and Cecile Garibay (2018) explain, such practices can be equitable and beneficial not only for the youth involved but also for the setting and field more widely.

The Bourdieusian-derived notion of STEM capital provides a useful conceptual umbrella that brings together STEM-related learning, knowledge and skills along with STEM-related attitudes, interests and behaviours. Bourdieu proposes that social life and practice is produced through the interaction of a person’s *habitus* (socialised and embodied dispositions that generate a ‘feel’ for the world and sense of self), *capital* (social, cultural, economic and symbolic resources) and the *field* (socio-spatial relations of power, which set the ‘rules of the game’ and determine the value accorded to youth habitus and capital). As we have previously detailed and discussed (Archer et al., 2015), from a Bourdieusian perspective STEM learning, knowledge, skills, attitudes and interests are all understood as forms of STEM capital, *the value of which is not fixed, but is determined by the field and shaped by interactions of structure and agency*. Similarly to the critical approaches, a Bourdieusian approach is concerned with issues of power and inequality and interrogates whose knowledges are valued and represented. Moreover, the Bourdieusian concept of dispositions, rather than attitudes, helps to convey the social and socialised (as opposed to purely individual cognitive) nature and ongoing re/production of young people’s feelings and views about science. In this way, the concept of STEM capital foregrounds an interplay of structure and agency within socialisation that produces layers of experiences that can generate a relatively enduring feel for whether STEM is ‘for me’, or not. As discussed by Archer and colleagues (2015), these dispositions can also be understood as forms of STEM capital. Moreover, the concept of STEM capital also encompasses STEM-related behaviours and social contacts and networks, which previous research has found to be importantly linked with youth engagement with STEM.

In this way, our model supports practitioners to recognise how equitable STEM capital outcomes for young people can be supported through the recognition and valuing of the STEM capital of underserved youth in ways that challenge and rework dominant notions of STEM, valuing and legitimizing minoritized STEM knowledges and resources.

## STEM trajectories

Many, but not all, ISL outcome frameworks try to capture outcomes relating to young people’s actual or intended continued participation and progression in STEM. These can include formal or informal educational participation and/or occupational routes. For instance, as the review led by Lloyd discussed:

A few [ISL practitioners] reported outcomes such as encouraging or preparing individuals for future STEM study, or for future STEM careers, as their main outcomes (ibid., p.5).

However, as Lloyd’s review also noted, where aspiration outcomes were captured, these tended to be short-term (e.g. measured immediately following an ISL experience), consequently, 'the extent to which these effects were sustained, and resulted in behavioural change in terms of STEM study or careers uptake, remains largely unknown' (ibid. p.12).

The critical literature explains how these traditional measures of progression can miss an understanding of the experience and process of progression for a young person, and specifically, the inequalities that will shape their trajectory. For instance, Tan and Calabrese Barton (2020) propose the term 'path hacking' as a way to capture how under-served youth have had to create their own pathways into STEM, often with improvised tools and in treacherous territory, because there were no pre-laid paths. These conceptualizations acknowledge how youth engage in practices that challenge and expand ways of being in STEM-related spaces and can help expand the future path-hacking possibilities for themselves and other youth. This literature also draws attention to how traditional linear notions of STEM progression do not acknowledge wider, multiple STEM pathway configurations, for instance beyond the so-called STEM ‘pipeline’. As discussed by Bobby Habig, Preeti Gupta, Brian Levine and Jennifer Adams (2020), progression outcomes from ISL can be particularly powerful when they impact beyond the confines of an ISL experience, to support trajectories in other fields, such as STEM routes in and beyond school and/or higher education.

In our model, we use the term STEM trajectories to refer to outcomes that support multiple forms of path-making and progression for under-served youth, both in and beyond STEM, in which STEM is the ‘vehicle’, not the ‘destination’. We use the term STEM trajectories as a shorthand term to capture aspects of path-making and progression, with the added sociological interpretation that the term trajectory conveys movement (with a direction and ‘speed’) that is acted upon by forces—helping to signal the role of structural identities and inequalities and contextual factors that can impact young people’s path-making and progression.

## STEM identity/STEM identity work

Most common, mainstream measures of ISL outcomes do not include items relating to STEM identity/STEM identity work, although some attitudinal measures may have some resonance of sorts with this outcome area (such as where they capture a young person’s interest in STEM or their feelings of STEM competence). However, the critical ISL and STEM education literature places considerable emphasis on the extent to which ISL participation might support young people’s identity work, including (but not limited to) a young person’s sense of themselves as a person who sees themselves and is recognised by others for their authentic use of STEM and being good at STEM. These ideas are explored in a range of studies, such as those by Calabrese Barton and Tan (2018), Jrene Rahm (2010) and Jennifer Adams and Preeti Gupta (2013). These studies underline how STEM identity outcomes are always provisional and in process. STEM identity (and the extent to which a young person is able to engage in STEM identity work) is understood as constructed and produced through a young person’s experiences in and interactions with the world, which are profoundly shaped by structural inequalities and power relations. Hence from a critical perspective, we are interested in the extent to which an ISL experience supports young people’s identity work both in relation to and beyond STEM. From this perspective, identity work is also intimately linked with other outcome areas, due to the foundational and mediating role of identity in relation to a young person’s learning, agency, trajectories and so on, as discussed more fully in work by Na'ilah Suad Nasir and Victoria Hand (2008).

The concept of habitus (which aligns with the concept of identity) is also central in Bourdieusian theory, with attention drawn to how social injustices are reproduced when the habitus and capital of oppressed and minoritized communities is devalued, ignored or is not recognised (or is misrecognized) within a field. That is, an equitable outcome would entail an ISL experience valuing young people for who they are and recognizing them as authentic and legitimate participants (with valued knowledge and expertise) within an ISL space.

Hence in our model, STEM identity work outcomes are understood as those that enable (1) meaningful identity work among under-served youth (self-recognition and recognition by others), in which their identities are valued as ways to engage with STEM and ISL, and (2) broadened STEM identity work among dominant youth participating in ISL who recognise the legitimacy and contributions of non-dominant communities as a result of the inclusion and valuing of under-served youth.

## Agency+ 

Most common ISL outcome frameworks do not capture outcomes relating to young people’s agency and more equitable forms of ISL representation and power relations. However, this theme is very prominent within the critical ISL literature, where emphasis is placed on expansive and consequential outcomes, but particularly where young people’s critical STEM agency is enhanced and dominant representations of STEM are reworked. For instance, Calabrese Barton and Tan (2020) identify how ISL experiences can support the development of critical STEM agency among minoritized youth, in which young people use STEM practices and knowledge to take action on issues that youth personally care about. Following the work of Megan Bang and Shirin Vossoughi (2016), equitable outcomes in this area also include the challenging and reworking of dominant STEM epistemological approaches and stances in ways that are more inclusive and valuing of diverse community and indigenous knowledges and forms of expertise. Such shifts, in turn, support the participation and voice of youth from under-served communities. Moreover, from a critical perspective drawing on the work of Bell et al. (2009), outcomes relating to agency and representation do not only occur at the level of the individual, but can also include collectively orientated outcomes, such as where ISL participation supports the disruption of wider, systemic injustices and/or increased community agency.

In our model, in the interests of short, convenient terminology, we use the shorthand term ‘Agency+ ’ to refer to outcomes related to agency, critical STEM agency and representation. Hence, Agency+ outcomes refer to the extent to which ISL participation results in: (1) supported and increased agency among youth, but particularly among underserved youth, (2) youth experiencing equitable participation and diverse and equitable representations of STEM and (3) underserved youth feeling that they have a rightful presence in ISL and STEM.

## Methods

In this paper, we apply the outcomes model to empirical data collected from ethnographic research conducted over the course of one academic year at four ISL settings in England, UK, as part of the wider Youth Equity + STEM (YESTEM) project on youth equity and informal STEM participation, funded by the Wellcome Trust, National Science Foundation and the Economic and Social Research Council. Two of the settings were based in the capital, London and two were located in Bristol, a city in the South West of England. The settings were selected as illustrative of a number of different ISL offers, including designed spaces (a community zoo and a science centre) and community spaces (a digital arts centre and a social enterprise working to support young women in STEM). We liaised with practitioners to identify a focal youth programme within each setting, namely a holiday programme at the zoo, a girls’ after-school STEM club, an after-school coding club at the digital arts centre and a youth engagement programme at the science centre. Together, these programmes offered us opportunities to consider youth outcomes across a range of activities, modes of engagement, ISL settings and young people.

### Data collection: youth ethnographies, observation and interviews

Multimodal youth ethnography work was conducted with 33 young people aged 11–14 who participated in programmes at the four partner ISE settings. Some were young people who were already naturalistically participating and others were recruited from under-represented communities to participate in programmes as part of the project. At three ISL settings, young people participated in distinct programmes and were recruited as part of this project: a weeklong programme at the community zoo, weekly school-based STEM club with two day trips as part of a girls-only STEM club, and bi-weekly school-based sessions with termly trips to the science centre. At the fourth ISE setting, young people were long-term participants, who had attended a range of different technology and media-focused programmes for between one and four years at the time of joining our study. Table 2 shows a summary of programmes and participants involved. Details of individual participants are presented in the Appendix. In addition, a young person’s self-identified ethnicity, gender and social class are given in the text the first time a young person is referred to in the paper. All young people chose their own pseudonyms.

**Table 2:** Overview of participating organisations, programmes and young people

Setting	Programme	Young people’s self-identifications	Nature of programme
Digital Arts Centre	Weekly technology-focused sessions and holiday programmes	6 boys 1 South Asian, 5 white British 3 working class, 3 middle-class	Weekly after-school drop in tech club that also runs during school holidays alongside holiday programmes. Participants code and create digital products (e.g. animations, programme robots)
Community Zoo	One-week holiday programme	4 boys, 5 girls 1 Black Caribbean, 1 Mixed South Asian /white European, 1 Mixed White European/North African, 1 Middle Eastern, 5 white British 4 working class, 5 middle class	One week, all day holiday programme. Participants learn about habitats, ecology, create portfolios, create artefacts for the zoo (e.g. bird boxes, signage, enrichment objects for enclosures) and participate in feeding animals and observing/recording and managing animal data (including some maths and statistics)
Girls STEM Club	Weekly school-based STEM club with industry visits (September–January)	10 girls 5 Black British, 1 Southeast Asian, 3 white British/Irish, 1 Mixed (Black Caribbean/White British) All working class	Weekly after school club with weekly themed sessions that showcase women in STEM (especially BAME) and have a carousel of STEM games and activities e.g. making and flying paper airplanes, modelling the solar system. Also includes a school visit industry day (learning to code an app) and a hackathon weekend (learning to programme in Scratch and Python)
Science Centre	Bi-weekly school-based club with visits to the science centre (one academic year)	4 boys, 5 girls 1 Black Caribbean, 1 Middle Eastern, 2 mixed race, 5 white British 3 working class, 9 middle-class	Young people are 'researchers' who contribute to the development of interactive displays in new exhibitions and are consulted by staff about the centre's programmes. Some sessions are held at school and a couple at the science centre (mixed STEM activities, e.g. making robots; discussion and development of exhibition ideas). Youth and their families are also invited to an open weekend at the science centre and each young person receives free passes for their family and friends to visit outside of the programme

Data collected included researchers’ observations of programmes; youth-constructed portfolio data (e.g. videos, photographs, written pieces of work, online posts, drawn pieces), audio-recorded interviews and discussion groups with young people and practitioners (at the start, during and after participation). In addition to the data collected during and immediately after the programme, we also met with young people c. six months after the end of each programme to ask more about the outcomes of their ISL participation. In the latter sessions, we used a set of outcome cards as group discussion prompts, based on the areas of the synthesised model, in which each card detailed potential outcomes relating to different outcome areas. For example, cards relating to the STEM trajectories included statements such as ‘The programme has made me want to do more STEM in the future’. Cards relating to STEM identity and identity work included statements such as ‘The programme has made no difference to how science-y I feel’. We worked with young people in small discussion groups of around three people, and asked them to look through the cards and discuss the extent to which they agreed or disagreed and their reasons, exploring differences and commonalities in view across the group. We also conducted individual follow up interviews, exploring participants’ experiences in greater depth.

### Data analysis

Analysis of the qualitative data followed several steps. First, following data anonymisation, a theory-led interrogation was undertaken. We constructed a ‘case’ for each young person, combining all the available data (e.g. artefacts, researcher observations, photographic evidence, practitioner accounts and all the different data produced by young people—including interviews, written and artistic products, and online data from their portfolios). As discussed further below, in a small number of instances, where there were ‘conflicts’ between the data, where applicable, primacy was given to a young person’s account and all examples of outcomes were recorded (e.g. a young person’s claim of a positive outcome on one occasion would still stand even if they claimed on a different occasion not to have experienced any positive outcomes sources). We then undertook deductive coding, using the synthesised model outcome areas identified in Table 1. Each youth case was coded for potential outcomes, which were identified and coded by two of the research team using the NVivo software package. Emerging codes were consolidated and refined through successive waves of coding and analysis, with researchers comparing coding and discussing discrepant cases in order to reach a shared understanding. Where codes were not easily resolved, a third member of the research team read the cases in question and a shared position was reached through discussion. This produced a set of data for each participant that was coded in relation to the five outcome areas in the model. For instance, data extracts that had been coded as examples of *grounded fun* characteristically included accounts of youth deriving pleasure, enjoyment and entertainment from participation and instances of young people laughing, smiling, examples of joyful playfulness and a sense of ‘buzz’ in a session. They also tended to involve some degree of ‘interest’ and intellectualised engagement and/or connection between the experience/content and the self within and alongside instances of enjoyment/entertainment.

We produced a set of 165 tables—five for each 33 youth participants, summarising data pertaining to five overarching outcome areas (Grounded fun, STEM capital, etc.), which we read across for each outcome area in turn (e.g. reading across all grounded fun tables), to arrive at an interpretation about how these outcomes were being achieved, or not, within and across the different programmes and participants. From these cross-case readings, we produced a memo, detailing key themes, for each of the outcome areas, which form the basis of the discussion of findings. Our data and our conceptualisation of outcomes mean that these cannot be ‘counted’ in any straightforward way—for instance, just counting the presence of an outcome can obscure the scale and nature of it, suggesting an equivalence between, for instance, a momentary, ‘slight’ outcome and a longer-term, more consequential outcome. However, to give some provisional sense of the ‘shape’ of our coding across the areas, we note here the *number of coded data extracts* that we found in relation to each area: Grounded fun (*n* = 31), STEM capital (*n* = 59), Science identity/work (*n* = 28), STEM trajectories (*n* = 30) and Agency+ (*n* = 28).

*Coding challenges: The complexity and inconsistency of outcomes.* We found the process of analysing data on potential youth ‘outcomes’ underlined how outcomes were rarely coherent or discrete in the data. For instance, different data sources sometimes pointed to different outcomes for a young person. While there were often overlaps and similarities across the data relating to either a particular youth and/or a particular setting, there were also multiple instances where practitioners, youth and researchers all identified different outcomes for a given youth at a particular time in a particular setting.

Take, for instance, Ginger (white, working-class boy, Digital Arts Centre). Ginger was regularly recognised as a coding expert both the practitioners and fellow participants during the club sessions. This recognition stood in stark contrast to his struggles for recognition at school. We recorded numerous examples in the observation and practitioner data that suggested Ginger experienced increased confidence and recognition as a result of his participation. For example, when asked what she thought Ginger gets out of coming to the weekly sessions, the practitioner leading the programme reflected 'it’s confidence, more than anything', recounting various examples of changes in his behaviours and interactions that she had seen over time. On a number of occasions, Ginger also concurred and talked about the skills, recognition and confidence that he had derived from participation. However, during his final follow-up interview, Ginger told us he was 'tired' and appeared to be in a more downbeat mood than when we had previously observed him. In this interview he voiced quite different views, saying that participation in the club had made 'no difference' to him, his confidence and his identification with STEM. He insisted instead that “no, it’s mainly school that made this happen”, attributing his coding interest and expertise to his first experiences of coding at school. Similarly, while Ginger had regularly attended club sessions that introduced new technical skills and content, in this particular interview he was adamant that he had not learned anything new from the programme ('Not really. I feel like I learn more from Mr H’s old YouTube channel').

So, how to account for these inconsistencies in the data? One potential interpretation is that Ginger did not like interviews and found it hard to represent himself verbally, so perhaps we should give less ‘weight’ to his verbal data compared to other sorts of data that we collected. Indeed, he had previously told us that he did not like sharing his views verbally and much preferred using digital technologies to express himself ('it’s quite hard for me to answer these questions […] when I type it I’m just so much better at it')—a point that we expand on elsewhere in relation to the importance of materiality in Ginger’s constructions of STEM identity (Godec et al., 2020).

From our conceptual position, we trust the voices and accounts of under-served young people (Mohanty, 2003). In doing so, we also recognise that there is no singular, consistent ‘truth’ of their views and experiences (in this case regarding what outcomes young people feel they derive from ISL). Rather, we respect that they will experience and voice multiple truths, each of which will be true to the moment and context in which they are expressed by a young person. Hence, we interpreted Ginger as telling us that there were numerous moments in which he felt that he derived valuable outcomes from his ISL participation, but there were also moments when he did not. On balance, his data tells us that he felt that he derived equitable outcomes from participation, but this was not absolute—there were also moments when he felt he had not derived value from the experience.

This pattern was also found across the other young people. For instance, some young people were much more positive about their experiences during the follow-up interviews than they had recounted during the actual sessions. For instance, 007 (white, middle-class boy, Science Centre) repeatedly shared his frustrations about the science centre programme with us during interviews and observations conducted during the sessions ('I feel like although we’ve done stuff, but we haven’t really done any stuff'; 'I found it a bit repetitive cause I think we’ve done it now three times now'; 'I’d like to do something more … bigger'). Yet, when we met him several months later, 007 enthusiastically identified a range of positive outcomes that he felt he had derived from his participation. For instance, in a card sort activity, he strongly agreed that he enjoyed the programme, learnt new things and gained new confidence through taking part.

In this way, we seek to embrace the complexity of young people’s experiences, recognising that they can and will often express contradictory views about the benefits they derive (or not) from their experiences. These accounts help us see how youth may articulate and experience different outcomes from their participation at different time points, and may feel differently about these outcomes in the moment during sessions compared to later follow-up interviews conducted several months afterwards. For instance, Emerald told immediately after the programme she found computing 'more interesting than before'. Yet when we caught up with her months later, she remarked that the programme did not make much difference, because 'I was always interested in it'. We suggest that both points need to be recognised and valued.

Others, like Ginger, above, were more negative in the catch-up interviews compared to previous data collection points. Young people who were interviewed at the end of an all-day session also tended to provide less positive accounts. In each case, we suggest that multiple interactions of personal, contextual, social and institutional factors will have shaped the accounts that they gave—there is no one ‘truth’, rather each account has a truth in the moment of its social construction.

These examples also draw attention to how young people’s ISL outcomes and experiences—and indeed their role in the research process itself—are not separate from their wider lives. That is, ISL outcomes, experiences and the research process are all situated within and mediated by wider identities and inequalities. There are many reasons why Ginger may have been tired on the day of his final interview, but it felt significant enough to him to voice it. Looking across his data, we interpret his feelings of tiredness as potentially mediating the extent to which he can derive equitable outcomes from his ISL participation. This leads us to consider how understanding and respecting how youth feel in the moment (and the extent to which these feelings may reflect or be exacerbated by wider relations and experiences of inequity in youths’ lives), and the extent to which ISL can enact relations of care in this respect, may be as, if not more, important for supporting equitable youth outcomes as the STEM content of a programme.

In the same way that we seek to privilege the views and experiences of young people from under-served communities, while recognising that these will often not be simple or consistent, we also wish to accord value and respect to the views of practitioners, noting that these will likewise reflect their own situated truths which may vary with time and context and which may, or may not, align with the young people’s accounts.

A further layer of complexity is added when we consider that each of the programmes also contained different component elements, again complicating the notion that there might be a generic set of outcomes that might be derived ‘overall’ from ISL participation. For instance, the girls STEM club included a regular after school STEM club, an industry visit and a weekend coding event. Young people, practitioners and researchers all associated different aspects of the programme (and indeed, different weekly club sessions) with different outcomes for different young people. For instance, Innocent (Black working-class girl) described some parts as ‘fun’ and ‘engaging’ but found other parts 'boring'. For instance, she described the day trip as 'pretty interesting but half of it, we already knew it. So, I had to sit there whilst they repeated it', whereas she described a club session that covered codes and maths games as 'all boring …the numbers, we already do like in Maths, so what's the point?'.

Matters were further complicated when young people did not always differentiate between the research component of the study (during which they worked with the research team to make their reflective portfolios and co-research their lives and ISE experiences, both on the programme in question and more broadly) and the STEM programme. For instance, Tori attributed a range of identity outcomes to the STEM club programme, but our analysis suggested that the experiences she described as producing these outcomes actually took place during research portfolio sessions, which were often held before or after club sessions.

This complexity, across and between data sources, participants and aspects of each programme, meant that there were no simple outcomes that could be identified from the young people’s ISL participation that could be straightforwardly and reliably measured. As explained, we see these variations as normal and to be expected—identities, experiences and outcomes are socially constructed and mediated phenomena that are generated through interactions between multiple actors across time and space. In this respect, we wish to strongly bracket the outcomes findings that we discuss next, noting that these are never neat or definitive, but are meaningful in their complexity. We have also intentionally foregrounded young people’s accounts of their outcomes, as opposed to researcher or practitioner interpretations, although at some points the latter are brought in where they seem to offer an additional dimension or potential interpretation for a point made by the young people.

## Grounded fun

Almost all the young people reported having enjoyed and had fun during their experiences on the programmes. The majority of young people were also observed by practitioners and researchers as enjoying themselves, for instance laughing and smiling, during the sessions. Whereas some traditional outcomes models might lead us to interpret these data as indicative of the programmes enabling the achievement of widespread fun outcomes among the participants, using a notion of grounded fun helps us to be more discerning, foregrounding only evidence of (1) youth having pleasurable, fun ISL experiences that they feel are grounded in their identities and what matters to them (2) youth having enjoyable experiences of ISL that challenge dominant normative STEM relationships and representations (as identified by either young people, practitioners or researchers).

The model focuses our attention on the extent to which young people from underserved communities had pleasurable experiences that were grounded in their identities and what matters to them in ways that challenged dominant STEM relations and forms of representation. We identified numerous examples of such outcomes, as exemplified by the case of Lulabelle, a White working-class girl who attended the zoo programme and who “loved” every aspect and moment of her participation, which both spoke to and extended her existing interest in animals and the environment and which—as discussed further in relation to Agency+ , built her self-confidence and developed her own critical eco-agency.

The importance of the fun being grounded is underlined by a number of instances in the data in which young people from under-served communities who experienced particular aspects of the programmes as being *just* fun (that is, not grounded in any way) were highly critical of these experiences. In these cases, fun was experienced as ‘hollow’, even boring. For instance, despite recognising the ‘fun’, playful elements of the girls STEM club programme, Innocent reflected “I’m not gonna lie, it’s boring”, which she explained as due to the fun not connecting with her own interest in science and learning, notably because she felt that the fun was not supporting her to learn anything important or new (“I wanna be learning something new”). Like several of her peers, she wanted more “real science” in the club sessions (by which she meant, new and meaningful knowledge and not replications of ‘recipe style’ school science experiments) and felt frustrated that her science knowledge and skills were not being extended. Similarly, Tori (Black working-class girl) found the sessions “fun” but at the same time wished there was “more science”, particularly “experiments”.

Privileged young people similarly felt that ISL participation had to be anchored in interest in order to be meaningful. As Spuggs and BnW (both White, middle-class boys) at the digital arts centre put it::

It’s fun. It’s not just something to do, it’s something *interesting* to do (Spuggs).

Like coding, it can be really tricky so you just don’t get it. So it’s interesting but not fun (BnW).

However, a number of young people from the Science Centre programme, only, or primarily, derived outcomes relating to momentary experiences of fun, with very little evidence of any wider outcomes or grounding in interest. They reported being “confused” by the purpose of the programme and struggled to articulate what they had got out of the sessions. For instance, Jack (White, working-class boy) described the sessions as “fun” but “confusing”. When asked what he thought the programme was about, he replied, “It’s just like a mystery. No one knows”. Although the young people all really liked the programme facilitator, Tessa (a White, middle-class woman), they also found the experience somewhat ‘hollow’ in its core content—a view that was also noted among more privileged young people on the programme, such as 007 (“I feel like although we’ve done stuff, but we haven’t really done any stuff”).

These interpretations lead us to question the received ISL wisdom that fun is an important hook that leads to engagement and wider outcomes. Indeed, we suggest that without a grounding in identity and interest, fun ISL experiences can be somewhat thin and inconsequential, even if they involve sociable and pleasurable experiences. In this way, the model helped us see how fun needed to be grounded if it is to be consequential.

In terms of the second aspect of grounded fun, we interpreted young people’s experiences of grounded fun as fun moments that involved some challenging of dominant narratives, e.g. the disruption of traditional, dominant ideas of STEM as being associated with Whiteness, masculinity and middle-classness (often epitomised in the notion of ‘cleverness’), challenging traditional notions of STEM hierarchy (e.g. adults as STEM ‘experts’ and youth as ‘not knowing’ about STEM) or as involved the valuing of youth community knowledges and expertise. We identified the most prevalent examples of where ‘fun’ disrupted dominant notions of science as elite and only for the ‘clever’ on the digital arts centre and zoo programmes, where the practitioners explicitly sought to disrupt traditional hierarchical relationships between educators and ‘learners’ and explicitly valued and foregrounded broader ways of doing and being in STEM (Archer et al., 2020). In the girls STEM club, young women commented on how much they liked the fun atmosphere that was created by practitioners (e.g. playing pop music, providing sweets and treats) which helped to challenge prototypical ideas of science learning, as involving hierarchical relationships and 'serious' learning. As Bubblepop (Black working class girl) told us, the sessions were 'more like a party' and Tori (Black working class girl) recounted “I found they [practitioners] quite upped the mood, 'cos I found they made jokes and stuff, which was really fun”. For Bubblepop and Tori (who had less detailed and extensive scientific knowledge and identification than Innocent), the fun was also grounded in that it supported them to 'learn new things'.

We interpreted the equitable potential of fun as constrained or negated when—despite using humour and spectacle—programmes reinforced notions of science and/or scientists as brainy and ‘mad’ (eccentric, zany). For instance, on the science centre programme one of the sessions involved the use of comedy props such as a large inflatable brain, reinforcing stereotypes of science and scientists as ‘brainy’. While most young people reported enjoying the session, we struggled to find any equitable outcomes for participating youth, which we interpret as exacerbated by missed opportunities to meaningfully ground fun during the programme.

Several of the practitioners also felt that fun needs to be grounded, for instance in Cole’s case, in wider outcomes, such as STEM learning. A couple of practitioners described how social inequalities mediated the extent to which fun might be desirable, achievable or appropriate. For instance, Kara (a White, middle-class practitioner in the science centre) noted that fun can be a 'luxury' that is easier for more privileged young people to experience in an ISL setting, due to intersectional injustices that position minoritized youth as ‘out of place’:

You can't have fun if you don't feel comfortable and you don't feel that you can enjoy [the experience] ... that's quite a luxury, fun, actually for a lot of young people.

Likewise, Kelvin (White working-class practitioner at the Community Zoo) remarked that although fun often acts as a 'hook' with many young people from more privileged backgrounds, in his work with young people on youth justice system programmes, fun was a trivial and even inappropriate outcome of little value or consequentiality, until more consequential outcomes (such as self-worth, identity and social skills) had been achieved:

Their first learning block is to be social and to work with others. Then they start to engage with STEM, and then they develop their own identity. The last thing we want them to do is have fun. I don’t want them to have fun while they’re with us. I want them to have a sense of self-worth. And then they have fun. (Kelvin, Community Zoo practitioner).

We also noted that even where a couple of underserved young people did *not* have fun on a programme, some still managed to achieve equitable outcomes—as in the case of Star (mixed White /North African, working class boy) who was recorded on numerous occasions during the Community Zoo programme as being grumpy or loudly complaining that he did not enjoy or like some of the activities, especially the outdoor sessions, those that involved contact with animals, creative tasks and those which involved group work (which he described as 'very frustrating … I prefer to just work by myself and do everything by myself'). However, while Star did not have fun, he did record consequential outcomes in relation to his STEM capital (e.g. telling the researchers a long list of things that he had learnt over the week, e.g. 'I didn't realise how many animals ate worms', 'I learnt about the different species of animals and I think animal taxonomy') and Agency+ (e.g. 'I went pescatarian!'). Moreover, a lack of fun did not seem to have any negative impact on his pre-existing STEM interests, identity and aspirations. We interpret Star’s case as exemplifying how fun needs to be grounded to support equitable outcomes, but also that fun alone is not necessarily related to, or required for, the achievement of equitable youth outcomes.

## STEM capital

Three of the programmes explicitly valued young people’s community knowledges and experiences and foregrounded these as legitimate ways of knowing within STEM, for instance regularly inviting young people to share their views, experiences and expertise and then recognising the value and authenticity of these as ways of ‘doing STEM’. We further noted that young people from under-served communities on these programmes were the most likely to report developing more positive views of and interest in science or technology as a result of their participation.

We also identified examples across all the programmes of young people from served and underserved communities reporting gaining new (canonical) STEM knowledge, understanding and skills, as well as some dispositional and behavioural changes and to a lesser extent new STEM-related social capital. For instance, most young people across the programmes recounted how they had learned new STEM knowledge and/or skills as a result of their experiences on the programmes. There were slightly different patterns between the settings, with STEM learning, expertise and skills outcomes being recorded most often in the Digital Arts Centre (particularly in relation to coding) and the Community Zoo (increased knowledge and understanding of animals, habitats and conservation and calculating and measuring skills). A number of youth across the programmes also reported more positive attitudes towards STEM as a result of their participation and a small number of youth, but notably those participating in the zoo programme, described having developed new STEM behaviours and hobbies as a result of participation, such as going outside and engaging with natural world more, (e.g. Iron, White working-class boy) and taking up bird watching (e.g. Tardis, Middle-Eastern, middle-class girl).

While all the programmes were successful in supporting STEM capital among young people who participated, the equitable outcomes model focuses particularly on the outcomes of underserved youth, because these have the potential to challenge inequitable social relations (supporting agency and wellbeing among under-served youth) and challenge dominant representations of who has, and gets recognised for having, STEM capital. The digital arts centre, community zoo and girls STEM club programmes recorded the most examples of underserved youth reporting STEM capital outcomes, as illustrated by the respective cases of Ginger, Lulabelle and Crystal, all White, working class young people. For instance, Ginger and Lulabelle described considerable new STEM learning as a result of participation in their respective programmes:

I now fully know how to use a Mac and a laptop. I used to get stuck a lot on what to do and then came here and now I can do loads of stuff […] I know how to use Photoshop and Illustrator and I can use Logic Pro and Garage Band. (Ginger, Digital Arts Centre)

I learnt also about the appropriate feed for animals and how they behave and also how animals respond to different behaviour. (Lulabelle, Community zoo)

Crystal, who attended the girls’ coding club, exemplified a range of STEM capital outcomes. In addition to new STEM learning and understanding across a range of topics and areas (including coding, the solar system and aerodynamics, to name but a few), Crystal reporting considerable changes in her view of science and STEM following her participation in the programme ('I used to absolutely hate Science and now I like it'). Crystal also seemed to develop new dispositions, such as recognising the transferability of STEM ('I learnt about different jobs involving science as well and what people did around science and technology').

A number of young people from the Digital Arts Centre, Community Zoo and Girls STEM club recounted talking more about STEM with family and community members as a result of participating in the programme. As Annie (White working class girl) explained, this marked a considerable change from previously, when she would 'never' have normally talked about science or STEM. This increase in talking about STEM at home also often involved the youth sharing their new expertise with family members and, as discussed next, gaining recognition from others.

## STEM identity work

The programmes all provided various support and spaces for young people to engage in STEM identity work, which included both a young person’s self-recognition (e.g. as being 'science-y', 'tech-y', a 'STEM person' or someone who knows/cares about science, tech or engineering) and receiving recognition from others (Carlone & Johnson, 2007). There were comparatively fewer examples of how the programmes were able to support young people to redefine dominant notions of what counts as being science-y or a STEM person. The equitable outcomes model focused our analytic attention specifically on the extent to which such identity outcomes were experienced by under-served young people, in order to foreground outcomes that are potentially more transformative (rather than reproductive) of dominant power relations.

On the whole, young people from under-served communities who participated in the ISL programmes were somewhat less likely than their White middle-class peers to express pre-existing STEM identifications. However, among those who did, we found examples of participation helping to reinforce these young people’s STEM identities and provide spaces for the practising of STEM identity work. For instance, in the girls STEM club, Bubblegum (White working-class girl) described herself as 'already science-y' and felt recognised by others as such. Innocent similarly explained how prior to participating she saw herself as a science person and someone who is 'good at science'. She felt that the programme may have helped reinforce this identity 'a little bit, but not much'.

We identified four young people (Ginger, Digital Arts centre; Crystal and Tori, Girls STEM club; and Lulabelle, Community zoo) for whom the programmes seemed to open up new forms of STEM identity work, specifically, shifts in coming to see oneself and be recognised by others as science-y or as a tech expert. For instance, as we discuss elsewhere in more detail (Godec et al., 2020), the digital arts centre programme supported Ginger’s STEM identity work and recognition for this identity work in more expansive ways that enabled him to see himself and be recognised by others for his tech and coding expertise that he did not experience as possible at school or at home, particularly as school forms of recognition were closely tied to narrow forms of academic attainment, from which Ginger was often excluded. In this respect, participation helped support Ginger’s own sense of being good at tech by recognising his tech expertise in ways that were not seen or recognised in the context of school technology classes. In this respect, the programme did not ‘change’ his tech identity, rather it provided a space of validation for Ginger’s tech expertise (supporting and valuing broader tech practices and forms of engagement than school) that resonated with Ginger’s own values and sense of identity.

In the girls STEM club, Tori (a Black working-class young woman) described how, as a result of her participation, she felt more personally confident with science and said that her classmates and teachers now recognised her as more science-y than before. Similarly, in her follow up interview, Crystal explained how she used to 'hate' both science and programming, but now enjoyed them more and felt that she understood science and programming better as a result of participation. She also said she saw herself as more science-y than before and that other people (notably family, friends and teachers) were starting to recognise her as more science-y and tech-y, due to her participation in the club. Likewise, in the Community zoo programme, Lulabelle described how the programme had helped her to feel more science-y and to gain recognition for not just academic performances of science, but specifically for 'hands on' and artistic/creative performances of science.

We interpret these examples as positive but also recognise that they did not necessarily reflect the majority of under-served young people across the programmes. Indeed, many of the young people who did not feel science-y before participating, told us that while they had enjoyed the programmes, they had not developed a sense of themselves as being science/STEM people nor could they identify any moments or examples when they had felt positioned as scientific (or STEM or tech experts) by others. For instance, Emerald (Black working-class girl) was adamant that participation had not changed her identification with science in any way and that her peers and teachers still did not recognise her as science-y. Annie expressed a very similar view and Innocent explained that despite the tech elements of the programme, she still did not feel at all tech-y in any way and could not imagine ever seeing herself, or being seen by others, as a tech person or good at tech.

While these findings might suggest a relatively limited impact on STEM identity outcomes across the programmes, as Bobbi (Black, working-class woman, head of the girls STEM club) noted during one of the collaborative research-practice reflection sessions (in which researchers and practitioners discussed emergent data and analyses), identity work is not 'one off', it needs to be repeated and consolidated over time. Hence, ‘changes’ in young people’s sense of self and the extent to which they are recognised by others will tend to be slow and require considerable resource, repetition and support over time before they might hope to become notable and sustained:

So I think [with] identity … time is important. Identity, I think, takes a lot longer ... For most girls, how they see themselves takes time, there needs to be some processing and decomposing. For example, even the girls who [now say] … ‘I didn’t realise women did this’ … for me, that’s what success looks like. … You can’t change how you see yourself overnight” (Bobbi, practitioner, Girls STEM Club).

A research study by Jennifer Langer-Osuna provides another possible interpretation of why so many young people suggested that they had experienced little or no change to their science/STEM identities. Langer-Osuna’s (2015) detailed case study of a Black American young man, Terrance, who took part in a mathematics programme, discusses how Terrance felt that while his actions changed over the course of the programme (notably becoming more engaged in the maths group work), this did not equate to a change in his identity ('Terrance claimed he had not changed “at all” while describing significant shifts in his own behavior and his orientation toward his in-school and out-of-school lives', p.78). In other words, he felt that his personal sense of identity had not been changed, even though his engagement did—a point that the author discusses as potentially reflecting his resistance of power and the efforts of those around him (such as teachers) to impose particular identities on him.

Indeed, we found comparatively more examples of the programmes developing new forms of STEM identity among privileged (White, middle-class) youth, hinting at the potential interpretation that dominantly configured STEM identities may be ‘closer’ (and hence less likely to evoke resistance) to the habitus/identity of privileged students. For instance, Spuggs, described how his participation in an ISL programme developed and fostered a new forms of STEM identity that he had not experienced before:

I don’t think I had any interest in tech before coming here. I used different tech often before coming, but I didn’t have much of an interest in it … I got more of an interest in science as well as tech, then realised that I like it and I realised I understood most of the things in science and that I could pick up on different things in science quite easily compared to other subjects. So I started doing better at science and then other people saw me more like a science person. (Spuggs, White, middle-class boy, Digital Arts Centre).

Similarly, BnW and Rob explained how participation in the programme helped reinforce their STEM identities, providing an additional spaces to perform pre-existing STEM identifications and identity work (e.g. 'I’ve always felt science-y', BnW; “' was already a tech person', Rob). While we recognise the inherent value of these identity work outcomes for the young people concerned, it is important to note that they are not inherently equitable. Indeed, we struggled to find examples within the data of more inclusive and transformatory STEM identity work among dominant youth participating in the four ISL programmes. Although as noted earlier, a number of them did record changes in their identification with STEM, either reinforcing and strengthening a sense of being a STEM person or developing a new self-recognition and/or recognition from others, we did not interpret these as specifically equitable outcomes because they were largely reproducing quite normative notions of STEM identity and did not exhibit any critical reflections on privilege or STEM. We also did not manage to identify any instances in our data of underserved young people’s identity work disrupting dominant representations of STEM, as has been noted in wider critical studies (for instance, youth redefining themselves as community science experts, as noted by Tan and Calabrese Barton, 2020), which would have been indicative of equitable STEM identity outcomes. As discussed further later, we interpret this as potentially reflecting how none of the programmes studied employed pedagogies that explicitly encouraged and supported youth to identify and recognise themselves in these more expansive and transformative ways—and that doing so might helpfully support the equitable potential of such programmes.

## STEM trajectories

Our analysis identified examples of how the programmes helped support changes in some of the young people’s STEM trajectories and (imagined) future participation in formal and/or informal STEM learning. As noted in relation to STEM identity, participation helped to reinforce the pre-existing STEM aspirations of a number of already “science-y”, middle-class young people, such as the technology-related aspirations of BnW and Spuggs (Digital Arts Centre) and on the community zoo programme, the animal-related aspirations of Charlie (mixed race middle-class girl) and Rhubarb (White middle-class girl) and the engineering aspirations of Ocean (White middle-class boy, Community Zoo). The equitable outcomes model, however, specifically foregrounds and values where this happens among under-served youth, such as White working-class youth like Ginger (Digital Arts Centre), and minoritized working-class youth like Innocent, Dani and Dinosaur at the Girls STEM Club, who all felt that participation had helped reinforce and support their existing STEM aspirations. For instance, Dinosaur (East Asian, working-class girl) felt that the STEM club had been helpful for her future and had made her “even more sure” that she wanted a STEM career.

While we found various examples of the programmes supporting pre-existing STEM aspirations, we found comparatively little evidence that participation had supported young people in their wider aspirations, both in and beyond STEM. For instance, only a couple of young people identified ways in which participation had supported their non-STEM aspirations more generally (largely in terms of supporting personal “confidence”, as Crystal put it) and most young people did not develop a new STEM aspiration as a result of participating, although several did suggest that their engagement had given them a new general interest in STEM which they would like to pursue through further participation in ISL settings. For instance, Annie (Girls STEM club) said that she would like to learn more about people in STEM even though she did not aspire to a STEM career (“Just to learn about it [STEM] because I probably wouldn’t want to do it in the future, but I would like to learn more about what STEM is and what we can do.”). Tori and Emerald (Girls STEM Club) also expressed a desire to participate in further STEM activities and ISL during the conversations that took place immediately after the end of the ISL programme (e.g. Tori said she “felt like going on a scratch programme” and Emerald wanted to “do more STEM”). However, follow up interviews a number of months later suggested that these intentions had not been realised (“to be honest, I kind of forgot about it. I just didn’t think about it”, Emerald).

An exception was Dragon, a mixed heritage (Black/White Irish working class) girl who attended the science centre programme. In a follow-up discussion, several months after the programme had ended, Dragon suggested that the programme had not only enabled her to get “more into science” but had also “made me want to do more of this in the future”:

Dragon: The programme has made me want to do more STEM stuff in the future. I’m not a really big fan of science and stuff, but this has made me more into science. It made me want to do more of this in the future

Interviewer: What kind of science might you do in the future?

Dragon: It has just opened up job choices as well. I thought science was boring, but now I can see it was fun. So, I can always take opportunities in science and engineering and stuff like that.

Although a relatively rare example in the data, we interpret this as a significant outcome, given Dragon’s position as a young woman from a community that is historically under-represented in STEM and ISL. The fact that Dragon articulated these views during the follow-up discussion group, several months after the programme, also hints at a relatively enduring outcome.

The model prompts us to remember that STEM aspirations and trajectories are not a primary goal or equitable outcome per se. Rather, an equitable trajectory outcome would be where an ISL programme supports the trajectories and path-making of a young person in relation to whatever areas they want to pursue (in or beyond STEM). That is, an equitable outcome entail STEM being the *vehicle* for supporting a young person’s trajectory, not necessarily the *destination* of their trajectory. In this respect, we suggest that the programmes examined had mixed results in terms of supporting young people’s trajectories in, beyond and through STEM.

## Agency+ 

In terms of Agency+ outcomes, we found a number of examples of young people’s agency being supported, particularly among underserved youth, most notable in terms of gains in personal confidence, environmental and/or socio-political agency. These outcomes were most prevalent in the Digital Arts Centre, Community Zoo and Girls STEM Club programmes. Most of these outcomes seemed to persist over time, in that they were still evident in follow up sessions, conducted several months later. As we now discuss, we found examples of both individual-level outcomes (predominantly supporting personal confidence) and more collectively orientated outcomes, which supported the agency and representation of under-served youth in ways that challenged dominant power relations.

Gains in personal confidence were reported by a range of young people (and noted by practitioners and researcher) across the programmes. This outcome was noted particularly among under-served young people, such as Ginger (digital arts centre), Crystal, Bubblegum, Tori (Girls STEM club), Magic (Black working-class boy, community zoo) and Lara (White European working-class girl, science centre). For instance, Magic reported gaining in confidence as a result of participation. Field notes also recorded changes in his participation over the programme, from being very quiet and seldom speaking at the start through to playing a more active role in discussions and offering his thoughts and views within activities. Likewise, Bubblegum described how she became more confident in herself and her social skills, which she attributed to her experiences of talking to new people and doing group work as part of the girls STEM club activities. Tori also recounted how doing group work with a girl she did not know during the industry visit helped make her more “bold and courageous” and that although she already felt confident in general, the programme had further increased her confidence (“I was already confident in myself but then that actually boosted it up more”).

One of the most notable gains in confidence was by Crystal, who was acutely shy and quiet when she joined the girls STEM club. Indeed, Crystal’s mother explained that her reason for consenting to participation was precisely because she hoped that the programme might help Crystal to become more confident in herself both personally and academically. Crystal gained in confidence and voice through her time participating—something that was also noticed and remarked on by her friends, teachers, family and researchers.

Because when I first started the class, I wasn't too sure about it and then after the first lesson I realised I really enjoyed it and so I enjoyed it more which made me more confident (Crystal).

Not all young people experienced the same outcomes and some, as epitomised by Innocent (Girls STEM Club), felt that participation had made “no difference” to her confidence (“I would say I’m already confident so it didn’t really change a thing about me”). We also noted similar outcomes among White, middle-class young people, like BnW, Rob and Spuggs who all felt that participation in the digital arts centre programme had built their self-confidence, a view that was reinforced by practitioners.

In addition to individual-level outcomes, we also noted some more collectively-orientated outcomes, as exemplified by a number of youth on the zoo programme, who reported what we term ‘critical eco-agency’. That is, they described having changed their environmental behaviours, exhibiting agency that enabled them to make more of a difference to their own lives and the world in general. Again, these gains were noted for both socially advantaged (e.g. Ocean, “I get to do things to help with animals, so it helps the environment as well, so like help them and promote them a bit more”) and under-served young people, with our attention being particularly focused on the latter. For instance, as a result of participating, Lulabelle explained how she had become more careful when recycling (“My recycling, I check what bin I put it in before I put it in the bin. … Anyway, so I recycle quite a lot now”). Iron explained that he now liked to go outside more than before. Star recounted how he was now taking better care of his cats and the environment as a result of the programme and had also become a pescatarian, as a result of the programme and discussions with the lead practitioner, Cole (mixed race, working-class man). We interpret these behavioural and attitudinal changes as examples of the young people developing critical STEM agency, which seeks to transform dominant relations of power and forms of representation.

We found examples across the programmes of young people having encountered diverse representations of ways of being and doing in STEM and of under-served young people feeling that they had a rightful presence in STEM as a result of their participation. These aspects were particularly strongly embedded in the rationale and practice on the digital arts centre and community zoo programmes. However, perhaps the clearest example of young people articulating these changes was in the girls STEM club programme. All the young women who took part in this programme, but particularly those from minority ethnic communities, reported being particularly inspired by the sessions in which they had learned about minority ethnic women scientists and engineers. For instance, Annie described how she now realised that women “can do science and can succeed in it too”. Dani (Black working class girl) reflected “I didn’t know much about women [in STEM] and [where I did] it was more white women than black women” and Innocent agreed “That was good to see because it’s usually, predominantly, a white career; if that makes sense?”.

Almost all of the young women who participated talked about feeling “empowered” by the regular foregrounding of Black and minority ethnic women STEM professionals. For instance, Emerald described how learning about women in STEM “encourages you and [helps] you think if they can do it that then you can do it too”. Similarly, Innocent explained that, as a young Black woman herself, although she already had high levels of personal confidence, finding out about Black women in STEM was “empowering for little girls like us, it makes me feel better”. The theme of agency (or empowerment, as the young women termed it) was echoed by Annie, who described feeling empowered by the knowledge that women are able to succeed as much as men.

You wouldn’t really listen [hear] about female mathematicians, it’s usually men, especially in the western world, so knowing that women can do it as well is really empowering to little girls like us, it makes me feel better (Innocent)

I think that it tells people that boys can't just do it, but girls can do it as well and [the girls STEM club] has taught me that (Bubblegum)

We interpreted these data as showing how issues of representation can be closely tied to a sense of rightful presence within a field, such as STEM and that supporting equitable outcomes of this type can be beneficial for all youth, but especially those from underserved communities.

## Discussion

In this paper we have attempted to grapple with the complex and slippery issue of how to understand and identify the outcomes that young people might derive from participating in ISL and specifically, those outcomes that might be considered equitable. The paper proposed a model of equitable youth outcomes that was applied to empirical data, collected with youth aged 11–14 who participated in one of four ISL programmes in two UK cities (London and Bristol). We discussed the complexity of identifying youth outcomes in the data and the often contradictory nature of data. We used the model to identify and explain examples of youth achieving equitable outcomes as a result of their participation in the programmes as well as examples of when these outcomes were not present. In this final discussion section, we reflect on affordances and limitations offered by the model and consider practical implications for the ISL sector with regard to supporting equitable youth outcomes from and through ISL.

We found the model useful for both helping us to both identify and understand the equitable potential of youth outcomes from ISL. We found the five areas of focus to be both workable and reasonably holistic, providing a framework that helped us foreground and identify outcomes that have the most equitable potential for under-served youth and challenging dominant power relations. From this perspective, and as we discuss further below, we suggest that ISL organisations and practitioners who are interested in issues of youth social justice, might find the outcomes model helpful for reflecting, planning and evaluating the equitable potential of youth ISL programmes.

Based on our analyses, we propose that greater recognition might usefully be given within traditional ISL research to how outcomes are highly complex and ‘messy’ phenomena—and that this point should be recognised conceptually, methodologically and empirically within ISL evaluation work. In this respect, we call for a move away from simplistic conceptualisations and measures of youth outcomes towards richer ways of engaging with outcomes that embrace the complexity and fluidity of these phenomena and which move beyond solely individually-orientated outcomes to also encompass those which are more collectively-orientated.

Our findings show that both dominant and under-served young people seemed to benefit from ISL participation and record a range of positive and desirable outcomes. However, the model helped us to ‘cut through’ the data to focus attention more purposively on outcomes with greater equitable potential. This helped to generate a number of insights relevant to research and practice. First, we suggest that the widespread emphasis that is currently placed on fun and STEM learning outcomes within ISL could be usefully reconsidered. Our analyses suggest that, from an equity perspective, fun is not necessarily required for the derivation of equitable outcomes and is of questionable value in the absence of a grounding in the identities and what matters to underserved youth. That is, fun needs to be grounded if it is to support consequential youth outcomes from ISL—otherwise fun ISL experiences can be hollow, or a luxury only for the more privileged. As noted by one of the practitioners, fun can be regarded as a luxury of the privileged if it is not grounded in the identities, values and needs of youth from under-served communities.

Second, many young people in our study seemed to derive some form of STEM capital as a result of their participation in the programmes, although the nature of this capital varied considerably, as did the equitable potential of acquiring STEM capital. Reflecting on our findings, we suggest that while most of the programmes did elicit and foreground the experiences and knowledges of under-served youth, there was scope to extend these practices further as a basis for supporting a more extensive and meaningful expansion of what and whose knowledge is legitimated as STEM, as argued by critical researchers such as Megan Bang and Shirin Vossoughi.

Third, while we found some evidence that ISL participation helped to reinforce the pre-existing STEM aspirations of both dominant and under-served youth participants, there was comparatively little evidence that ISL participation had supported ‘non-STEM’ youth to develop new STEM trajectories. Given that wider critical work has found that, given the appropriate pedagogy and resources, the STEM trajectories of under-served youth can be supported, we interpret our data as suggesting that this needs to be a more explicit focus of the pedagogy and practice of the programmes we studied, in order to enhance this outcome area further. In particular, under-served young people experienced blocks and challenges to their STEM trajectories and continued participation once programmes had ended. This might be a useful area for further joint research-practice reflection, asking how might under-served young people’s STEM trajectories be supported and bridged once they are no longer participating in particular ISL programmes?

Fourth, we interpret our findings as underscoring that supporting young people’s identities and trajectories takes time and resource. On the whole, the programmes appeared to be more effective in reinforcing pre-existing STEM identifications and aspirations among young people (from both privileged and under-served communities) with comparatively fewer instances of participation supporting wider identity work and/or aspirations. While we do not consider an aim of ISL being to ‘convert’ young people to STEM identities and trajectories, in light of claims made for the potential of ISL to support more diverse participation in STEM, we found some examples of the programmes supporting under-served young people to be recognised for their STEM expertise and in enabling young people to find interest and meaning in and through STEM when it was more inclusively configured. We did identify three instances in which consequential and equitable shifts seemed to occur for particular young women from underserved communities. We interpret these findings as suggesting that ISL programmes can usefully support the ‘non-STEM’ identity work and trajectories of young people who do not feel that STEM is ‘for them’, especially among those from under-served communities. Moreover, there is scope for further efforts to ensure that the identity outcomes associated with a programme are not solely located at the level of individuals from under-served communities—practitioners and socially privileged youth need to also recognise and value the legitimacy and contributions of youth from under-served communities. These sorts of shifts in power, practice and representation take time and we suggest that programmes, such as those we studied in the community zoo, girls STEM club and science centre, could usefully shift towards longer-term ways of working with underserved youth in order to further build an ethos of social justice within the setting and to support equitable identity outcomes for all youth. We also suggest that more emphasis and consideration might be usefully given to supporting more equitable and transformatory identity outcomes among dominant youth—that is, to enable them to engage in more expansive and critically aware STEM identity work that is informed by the identities and experiences of under-served youth, and which disrupt rather than reproduce dominant and elitist forms of STEM identity.

Finally, we identified various examples of equitably consequential outcomes for youth in relation to agency and representation. While we do not dispute the importance and value of individually orientated outcomes, such as increases in personal confidence, we were particularly interested in more collectively-orientated outcomes that related to enhanced agency among under-served youth, through the development of critical eco-agency (on the zoo programme) and in relation to the recognition and rightful presence of Black and minority ethnic women in STEM among young women on the girls STEM club programme. We suggest these are not always common foci on traditional ISL programmes and evaluations and would seem to offer useful avenues for further exploration.

The model was designed as a practical tool to help support ISL practice, but we suggest that it may also have a role to play as a resource that can support ongoing efforts to re-imagine of ISL in more socially just ways and help support the practice of educators, who—as a long history of existing research underlines—may wittingly or unwittingly reproduce injustices through their practice (as epitomised by the extensive work of Mary Atwater and colleagues, such as Atwater 2000). For instance, the model can be seen as a practical tool to help foreground questions such as whose identities, knowledges and ways of knowing are being recognised and legitimated in and through ISL programmes? It helps trouble the primacy that is traditionally given within ISL outcome measures to dominant forms of STEM knowledge and learning and it prompts us to question some of the common assumptions and rationales for ISL, suggesting that the value and purpose of ISL lies in its capacity to be a vehicle for social justice, not in servicing the STEM ‘pipeline’ and the continued dominance of socially privileged communities and the Global North. In particular, the model adds weight to existing arguments regarding the importance of foregrounding equity in central and intentional ways within STEM—because not doing so is not neutral, but rather entails the reproduction of injustices.

## Using the model in ISL practice

We have distilled insights derived from the application of the model to empirical data into Table 3, which provides some suggested ways that the model might be operationalised in practice—detailing some key ways in which outcomes might be measured as part of ISL evaluation and what evidence might usefully be collected in support.

**Table 3:** Operationalising the ISL Youth Equitable Outcomes model in ISL evaluation and practice

Outcome area	Guiding Questions	Example evidence
Grounded fun	To what extent are young people’s pleasurable/fun experiences of ISL (1) connected to (grounded in) their identities and what matters to them? (2) challenging dominant normative STEM relationships and representations?	Young people reporting that ISL experiences are interesting and engage with issues that are important to them Fun activities and representations of science/scientists are inclusive and diverse and grounded in marginalized youths’ lives and what matters to them
STEM capital	To what extent is the STEM capital of underserved youth being valued, supported and augmented?	Examples of how under-served young people’s community knowledges are valued and drawn on in ISL Programmes engage meaningfully with questioning whose knowledge and expertise is being recognised in and through STEM Evidence of under-served youth gaining meaningful STEM knowledge/skills, STEM social contacts and networks, dispositions and everyday STEM engagement Evidence that the expertise of under-served youth is being leveraged, valued and recognised in and beyond ISL
STEM identity/work	In what ways does the ISL experience support young people to: (1) (re)define what and who counts in STEM? (2) recognise the community and STEM expertise of under-served young people? (2) feel valued and recognised by others for their (STEM) expertise? In what ways does the ISL experience support privileged youth to (1) understand and recognise inequalities in STEM and (2) be more reflexive and inclusive	Evidence that young people feel engaged, enabled, and empowered within the ISL programme/activity to have on going dialogues about their identity and what matters to them Evidence that support for young people’s STEM identity work is systemic and embedded in ISL Evidence of programmes valuing young people for who they are, not who they are expected to be
STEM trajectories	How are under-served young people’s trajectories and path-making/progression being supported and enhanced within, beyond and through ISL/STEM?	Examples of under-served youth being supported in their trajectories both in and beyond STEM
Agency+	How is the ISL experience (1) supporting young people’s agency, but particularly among underserved youth? (2) actively challenging unjust STEM representations and practices? (3) enabling under-served youth to have a rightful presence and feel that they belong in ISL and STEM?	Examples of young people using their (STEM) expertise to take action on issues that matter to them Examples of young people redefining STEM in more equitable and inclusive ways—and evidence of these redefinitions being recognised and valued by others (particularly educators, adults, peers and dominant others) Evidence that under-served young people feel a sense of belonging and ownership in ISL and STEM and feel that people like them are well represented, valued and present in these spaces Evidence that young people’s wider lives, opportunities and well-being have been positively impacted by an ISL experience

### Limitations

We found that the analytic process was, perhaps inevitably, quite labour intensive and required considerable interpretation. Applying the model to our data was not quick or easy—features that may often be desirable within ISL evaluation research. The model is also limited to *youth* level outcomes and does not cover practitioner and/or institutional outcomes and those relating to the wider fields of STEM and ISL, although these are the subject of forthcoming work.

Largely the outcomes we evidenced in this paper were limited to the ISL programme spaces and we did not gather data relating to potential wider outcomes, such as those relating to young people’s wider home or school lives, even though we recognise the importance and consequentiality of these wider outcomes. We were also limited by the relatively short-term nature of the outcomes data collected, up to six months after the young people’s ISL participation. Ideally we would have had the time and resource to extend data collection further.

We acknowledge that the findings reported in this paper are based on small and unrepresentative samples of students participating in just four programmes in two cities in England and hence cannot be generalised to the ISL sector more widely either nationally or internationally. However, we hope that they might provide interesting and potentially useful pointers for both further research and to support equitable practice.

## Conclusion

This paper has sought to add to add new insights to the complex challenge of how to identify the outcomes that young people might derive from participating in ISL, but specifically, those outcomes that might be considered equitable. The paper proposed a conceptually driven model (developed from critical ISL and sociological theory and research) that included five aspects of equitable youth outcomes (grounded fun; STEM capital; STEM identity work; STEM trajectories; Agency+) that was applied to empirical data, collected with 33 young people aged 11–14 who participated in one of four ISL programmes. The complex and contradictory nature of outcomes data were discussed and findings were presented detailing a range of examples of youth achieving equitable outcomes as part of their participation in the programmes—along with examples of when these were not present. In conclusion, we suggest that the model offers some new ideas and a tool for ISL research and reflective practice to help support equitable youth outcomes from and through ISL participation.

## Appendix: Demographics of youth participants

**Table:**

Pseudonym	ISL setting	Gender (self-identified)	Ethnicity (self-identified)	Social class (parental occupation/Free School Meals)
Lulabelle	Community Zoo	F	White British	Working class
Magic	Community Zoo	M	Black Caribbean	Working class
Evie	Community Zoo	F	White British	Middle class
Charlie	Community Zoo	F	Mixed race	Middle class
Rhubarb	Community Zoo	F	White British	Middle class
Iron	Community Zoo	M	White British	Working class
Ocean	Community Zoo	M	White British	Middle class
Star	Community Zoo	M		
Mixed (White European/North African)	Working class			
Tardis	Community Zoo	F	Middle Eastern	Middle class
Black-and-White (BnW)	Digital Arts Centre	M	White British	Middle class
Rob	Digital Arts Centre	M	White British	Middle class
Triangle	Digital Arts Centre	M	White British	Working class
Spuggs	Digital Arts Centre	M	White British	Middle class
Ginger	Digital Arts Centre	M	White British	Working class
Beast	Digital Arts Centre	M	South Asian	Working class
Tori	Girls STEM Club	F	Black British	Working class
Innocent	Girls STEM Club	F	Black African	Working class
Dani	Girls STEM Club	F	Black African	Working class
Crystal	Girls STEM Club	F	White British/Irish	Working class
Emerald	Girls STEM Club	F	Black African	Working class
Dinosaur	Girls STEM Club	F	East Asian	Working class
Bubblepop	Girls STEM Club	F	Black African	Working class
Avette	Girls STEM Club	F	Mixed (Black Caribbean/White)	Working class
Annie	Girls STEM Club	F	White British	Working class
Bubblegum	Girls STEM Club	F	White British	Working class
Fox	Science Centre	F	White British	Working class
00 7	Science Centre	M	White British	Middle class
Reek	Science Centre	M	White British	Working class
Dragon	Science Centre	F		
Mixed (Black Caribbean/White British)	Working class			
Unicorn	Science Centre	F	White British	Working class
Lara	Science Centre	F	White European	Working class
Wolf	Science Centre	M	Mixed (Middle Eastern White European)	Working class
Jack	Science Centre	M	White British	Working class
